# Antibiofilm Activity of Plant Polyphenols

**DOI:** 10.3390/molecules21121717

**Published:** 2016-12-13

**Authors:** Lívia Slobodníková, Silvia Fialová, Katarína Rendeková, Ján Kováč, Pavel Mučaji

**Affiliations:** 1 Institute of Microbiology of the Medical Faculty and the University Hospital in Bratislava, Comenius University in Bratislava, 811 08 Bratislava, Slovakia; livia.slobodnikova@fmed.uniba.sk; 2 Department of Pharmacognosy and Botany, Faculty of Pharmacy, Comenius University in Bratislava, 832 32 Bratislava, Slovakia; rendekova18@uniba.sk (K.R.); mucaji@fpharm.uniba.sk (P.M.); 3 Department of Stomatology and Maxillofacial Surgery of the Medical Faculty and the University Hospital in Bratislava, Comenius University in Bratislava, 812 50 Bratislava, Slovakia; mudr.jan.kovac@gmail.com

**Keywords:** plant polyphenols, antibacterial activity, bacterial biofilm, medical device-associated infection, dental caries, periodontal disease

## Abstract

In the history of human medicine, antibiotics represent epochal examples of medical progress. However, with an approaching antibiotic crisis due to the emergence and extensive spread of antimicrobial resistance among bacterial agents, as well as to increasing number of patients with chronic and recalcitrant bacterial biofilm-associated infections, the naturally occurring molecules may become new sources of antibacterial and antibiofilm drugs for clinical usage. Polyphenols represent a class of plant natural products which are important in plant defense against microbial pathogens. The main focus of the review is on the antibiofilm activities of phenolic compounds against bacteria which play an essential role in medical device biofilm-associated infections. The other, not negligible part of the review is devoted to polyphenols’ activity against bacterial agents that cause dental caries and periodontal disease.

## 1. Introduction

The introduction of antibiotics to clinical practice represents one of the most outstanding contributions to the treatment of life-threatening infectious diseases. However, due to the extensive use of these valuable therapeutics, numerous resistance mechanisms have emerged and rapidly spread among bacterial disease-causative agents. Modern medicine is nowadays facing the threat of returning to the pre-antibiotic era, at least for some types of infectious diseases. The rapid spread of multidrug-resistant (MDR) or extremely drug-resistant (XDR) bacterial strains seems to be the most frightening development. Along with some community strains of *Mycobacterium tuberculosis*, *Streptococcus pneumoniae*, or *Neisseria gonorrhoeae*, these MDR and XDR bacterial strains are represented mainly by nosocomial opportunistic bacterial infectious agents, such as methicillin resistant staphylococci (*Staphylococcus aureus* and coagulase-negative staphylococci), vancomycin-resistant enterococci, and the Gram-negative XDR strains. Great concern has arisen due to the appearance and spread of MDR and XDR strains of *Pseudomonas aeruginosa*, *Acinetobacter* spp., and carbapenem-resistant *Enterobacteriaceae* (mostly contributed by *Klebsiella pneumoniae*) [1,2].

In addition the abovementioned resistance caused by mutations or resistance gene acquisition, the second large challenge is represented by the phenomenon of recalcitrant infections in patients with bacteria or fungi growing in biofilms on implanted or inserted medical devices, or in the tissue damaged by various prior pathological processes [3]. A distinct subset of such infections is represented by dental caries and periodontal diseases, caused by members of the normal oral microbiota [4,5].

A biofilm is a sessile form of bacterial existence on solid surfaces or air-liquid interfaces, in which bacteria multiply covered by a self-produced biofilm matrix, composed of bacterial intercellular polysaccharides, proteins, and extracellularly released nucleic acids [6]. The protective effect of bacterial biofilm phenotypes is multifactorial. It includes decreased penetration of antimicrobial agents into the deep layers of biofilms, the capture of positively charged molecules by the extracellular polymeric biofilm matrix, or the ability of biofilm matrices to concentrate bacterial enzymes which inactivate antibiotics [7]. Gradients of nutrients, metabolites, oxygen, pH, redox potential, or antibiotics penetrating to the biofilm produce an environmental stress in the bacteria, resulting in the expression of inducible resistance mechanisms, increased mutability rate, and bacterial adaptive phenotype changes. These changes lead to metabolic suppression in bacteria, which cause increased ability to survive exposure to antibiotics and an increasing rate of persister cell formation. Bacterial persisters survive antimicrobial therapy and may reseed the patient’s infectious focus after discontinuation of antimicrobial therapy, resulting in disease relapse [7,8]. Mechanisms of bacterial adherence, biofilm accumulation, and bacterial dispersion from the mature biofilm, coordinated by quorum-sensing (QS) chemical signals of inter-bacterial communication, reflecting the bacterial population cell density play a vitally important role in the process of biofilm development [9].

Therefore, together with the steps taken to reduce the threat of an antibiotic resistance crisis and the search for new antimicrobial agents [10], research on agents able to modulate some important virulence factors of bacteria, such as adhesivity, biofilm formation and the phenomenon of bacterial persistence, has an exceptional importance for the development of new therapeutics for medical practice.

Many reports on antibacterial activity associated with extracts from an enormous range of plants can be found in the literature. The discovery of novel antibacterial agents in plant extracts most frequently begins with leaves or roots from healthy specimens, even though there is ample evidence that many key components of plant defenses against phytopathogens are induced by infection. Plants respond to microbial attack through a highly coordinated repertoire of molecular, cellular and tissue-based defensive barriers to colonisation and invasion [11,12,13]. Plant secondary metabolites, among them many polyphenols, such as flavonoids, phenolic acids, and tannins, show antibacterial and/or antibiofilm activities. This review focuses on the antibiofilm activities of the abovementioned plant products.

## 2. Main Antibiofilm Phenolic Compounds

Plant polyphenols represent a large class of biologically active secondary metabolites of plants. They include flavonoids, tannins, anthocyanins, phenolic acids, stilbenes, coumarins, lignans, and lignins [14]. These substances play an important role in resistance against various microbial pathogens and protect against free radicals and toxins [15,16]. Nowadays, plant polyphenols enjoy an ever-increasing recognition not only by the scientific community but also, and most remarkably, by the general public because of their presence and abundance in fruits, seeds, vegetables, and derived foodstuffs and beverages, whose regular consumption has been claimed to be beneficial for human health. They have often been highlighted due to their capacity to scavenge oxidatively generated free radicals that underlies their utility in reducing the risk of certain age-related degenerative processes and diseases [16].

In phenolics, multiple mechanisms of antibacterial activity have been described: they interact with bacterial proteins and cell wall structures, they may cause damage to cytoplasmic membranes, reduce membrane fluidity, inhibit nucleic acid synthesis, cell wall synthesis, or energy metabolism [15,17,18]. On the other hand, antibiofilm activity research on plant phenolics has revealed, besides their destructive activity on bacteria, also “softer” activities leading to biofilm suppression by affecting the bacterial regulatory mechanisms such as quorum sensing or other global regulator systems, without an effect on bacterial growth [19]. A survey of recently published antibiofilm activities of flavonoids, phenolic acids and tannins is provided in Table 1.

To the most extensively studied bacteria from the point of view of biofilm production belong *Staphylococcus aureus* and coagulase-negative staphylococci, which play a crucial role in medical device-associated infections [52], and one of the most important dental caries agents—*Streptococcus mutans* [4].

Tannins represent one of the biggest groups of plant polyphenols. They are subclassified into condensed tannins (proanthocyanidins or catechins) and hydrolysable tannins (gallotannins and ellagitannins) [53]. Gallotannins and ellagitannins derived from the metabolism of the shikimate-derived gallic acid (3,4,5-trihydroxybenzoic acid) result through various esterification and phenolic oxidative coupling reactions in yield numerous monomeric, oligomeric and polyphenolic galloyl ester derivatives of sugar, mainly d-glucose [54].

**Figure 1:**
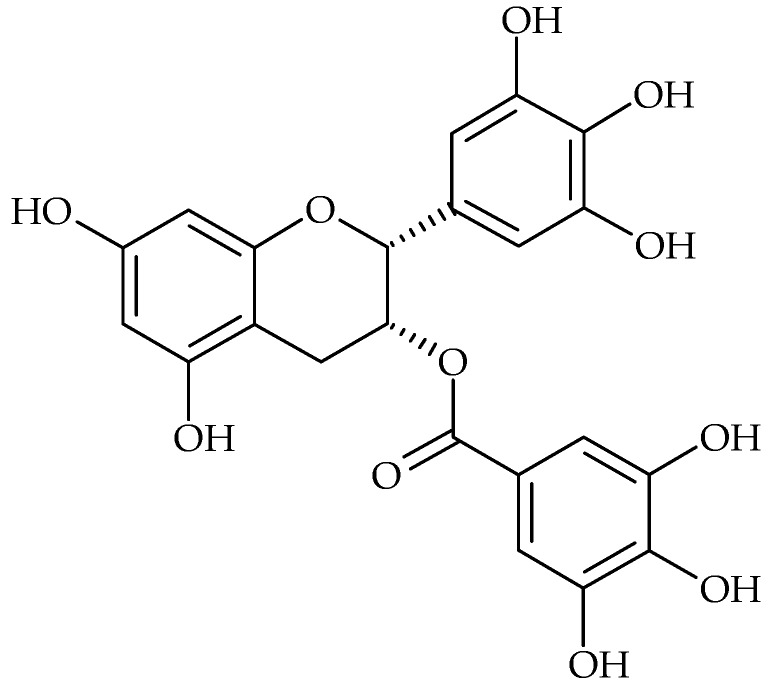
Chemical structure of (−)-epigallocatechin gallate.

Tannins possess antibacterial activity both against Gram-positive and Gram-negative bacteria. For example, the catechins are able to penetrate and interact with lipid bilayers [55]. Alternatively, they may cause membrane fusion, a process that results in leakage of intramembranous materials and aggregation [56]. Green tea (*Camellia sinensis*) rich in catechins has the capacity to reverse methicillin resistance in MRSA isolates at concentrations much lower than those needed to produce inhibition of bacterial growth [57]. Roccaro et al. referred to the modulation effect of catechin gallates to bacterial drug resistance. It has been shown that epigallocatechin gallate (EGCg) (Figure 1) had several antibacterial activities, limiting bacterial growth and invasion and acting in synergy with some antibiotics. Sub-inhibitory concentrations of EGCg were able to reverse tetracycline resistance in staphylococci by inhibition of the Tet(K) efflux pump, in addition to further sensitizing of the susceptible staphylococcal isolates to this antibiotic [58]. Concerning *S. aureus* biofilm formation, EGCg at subinhibitory concentrations has shown to decrease slime production, therefore inhibiting biofilm formation by this bacterial species [44].

Tannic acid from black tea (*Camellia sinensis*) inhibited *S. aureus* biofilm formation without inhibiting bacterial growth via a mechanism dependent upon the putative transglycosylase IsaA, and this acid also inhibited pharyngeal colonization with *S. aureus* in an in vivo rodent model [59]. Extract of *Alnus japonica*, with quercetin and tannic acid as the major anti-*S. aureus* biofilm compounds, was the most active from 498 screened plant extracts. It inhibited biofilm formation by influencing the expression of genes linked to biofilm production, most markedly *icaA* and *icaD* [60].

*S. aureus* antibiofilm activity was described in several phenolic acids, including gallic [61], ellagic [62], ginkgolic [63] and rosmarinic acid [49] at subinhibitory concentrations.

**Figure 2:**
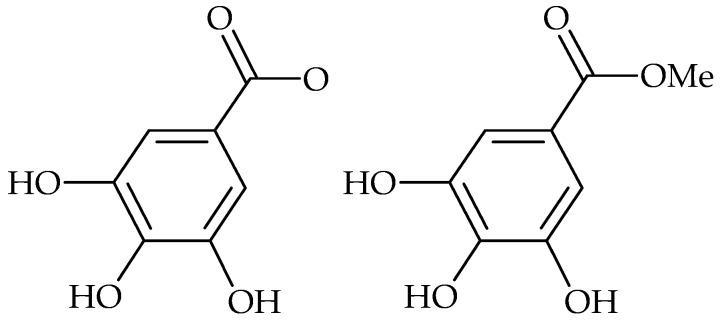
Chemical structures of gallic acid (**left**) and methyl gallate (**right**).

Recent research on *Cotinus coggygria* leaves rich in gallotannins such as gallic acid and methyl gallate (Figure 2), showed a good activity against *S. aureus* in planktonic and biofilm growth forms. The 60% methanol extract showed bactericidal activity against all tested *S. aureus* strains, including polyresistant strains, and eradicated bacteria in already established 24-h biofilm [43].

**Figure 3:**
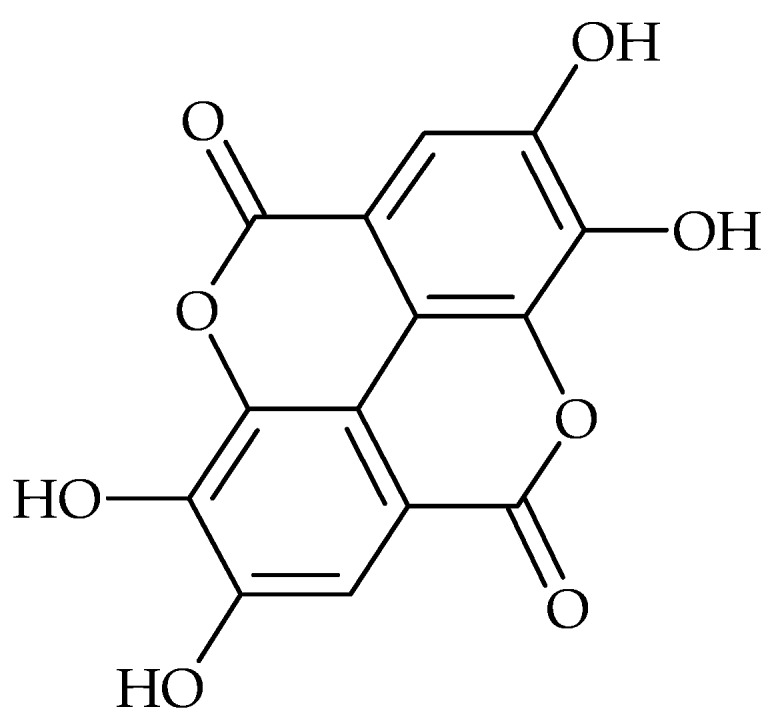
Chemical structure of ellagic acid.

Ellagic acid and its derivatives from *Rubus ulmifolius* can limit *S. aureus* biofilm formation to a degree that can be correlated with increased antibiotic susceptibility [64]. Ellagic acid (Figure 3) and tannic acid were also tested for their ability to inhibit biofilm formation by *Escherichia coli*. Both compounds reduced biofilm formation significantly. However, no synergistic effect of these two compounds was observed [48]. Methanol extract of pomegranate, rich in ellagic acid, was also shown to inhibit the formation of biofilms of *S. aureus*, methicillin resistant *S. aureus* and *E. coli* as a result of possible damage to the cell membrane [62].

Inhibition of biofilm formation on surfaces covered by plant products may be significant in the future techniques which prevent medical device biofilm-associated infections. Such activity was described in several studies on the antibiofilm activity of plant polyphenols.

**Figure 4:**
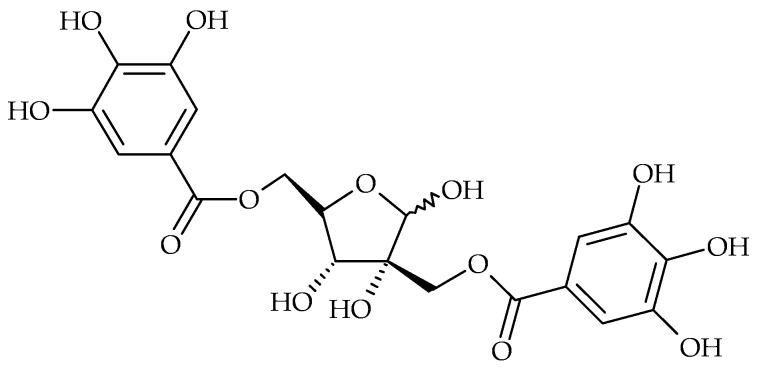
Chemical structure of hamamelitannin.

Tannic acid (syn. gallotanin) from *Eustigma oblongifolium* inhibited biofilm formation by *S. aureus* independently of growth mechanisms. It prevented the initial attachment to solid surfaces and the synthesis of polysaccharide intercellular adhesion compounds. The antibiofilm activity of gallotanin was expressed after application in solution, as well as after coating of the tested surfaces [51]. Similar effect had medical device implant surface coating with hamamelitannin (2′,5-di-*O*-galloyl-hamamelose, Figure 4) isolated from the bark and leaves of *Hamamelis virginiana*. Medical device-associated infection in rat model was completely prevented, when sterile collagen-sealed double velour-knitted polyethylene terephthalate (Dacron) grafts were coated with hamamelitannin. No activity of hamamelitanin on bacterial growth was observed and the antibiofilm activity was attributed to the staphylococcal quorum-sensing regulator RNAIII inhibition [65].

Trentin et al. reported that B-type linked proanthocyanidin-coated surfaces reduced *S. aureus* and *E. faecalis* adhesion. The proposed mechanism of bacterial attachment inhibition is based on electrostatic repulsion, high hydrophilicity and the steric hindrance provided by the coating that blocks bacterium-substratum interactions [66].

**Figure 5:**
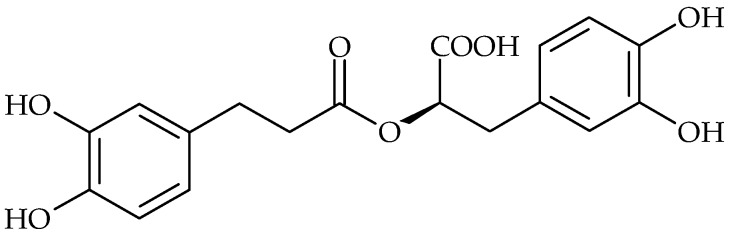
Chemical structure of rosmarinic acid.

Rosmarinic acid (Figure 5), also known as Lamiaceae tanning compound, was identified as a major phenolic compound in many antimicrobially active plants, e.g., in the genera *Mentha, Melissa, Lycopus, Origanum, Thymus, Salvia* [67]. According to the latest research, rosmarinic acid could be a candidate topical antimicrobial agent with killing activity on planktonic forms of clinical *S. aureus* strains and suppressing activity in the early stages of biofilm development [49,50]. At subinhibitory concentrations near to MIC this compound suppressed *S. aureus* biofilm production; however, with further decreases of the rosmarinic acid concentration an increase of biofilm production was observed, which reached its peak at 100-times lower concentrations than MIC [49]. A similar phenomenon of concentration-dependent response in biofilm production was observed in the case of many other antimicrobial agents, as an expression of bacterial stress response modulated by low concentrations of chemical compounds, such as ethanol or antibiotics [68,69,70]. Therefore, the phenomenon described above should be tested and considered when determining the therapeutic concentrations of the potential drugs of plant origin, as underdosing might have counterproductive effects on biofilm-related infections.

**Figure 6:**
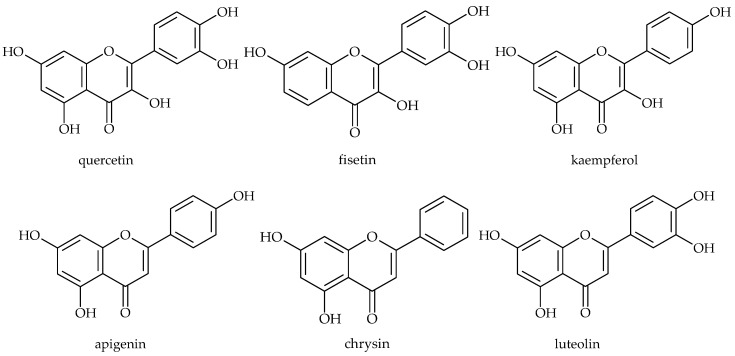
Chemical structures of red wine components: quercetin, fisetin, kaempeferol, apigenin, chrysin and luteolin.

Flavonoids are widely distributed phenolics characterized by a phenylbenzopyran chemical structure. In plants, flavonoids have long been known to be synthesized in specific sites and are responsible for the colour and aroma of flowers and fruits to attract pollinators, and consequently fruit dispersion animals; they help in seeding, germination, growth and development of seedlings. Flavonoids protect plants from different biotic and abiotic stresses and act as unique UV-filters. Flavonoids have roles against frost hardiness, drought resistance and may play a functional role in plant heat acclimation and freezing tolerance. They function as signal molecules, allelopathic compounds, phytoalexins, detoxifying agents, and antimicrobial defensive compounds [54,71]. Three different modes of antibacterial activity of flavonoids were described in the literature. The first corresponds to nucleic acid synthesis inhibition [72]. The second way involves damage of the cytoplasmic membrane by a perforation mechanism [73] and a decrease in membrane fluidity [74], and the third, the inhibition of energy metabolism [75]. Flavonoids also exhibit antibiofilm activities. Red wine (from *Vitis vinifera*) contains, besides tannic acid and *trans*-resveratrol, plenty of flavonoids such as quercetin, fisetin, kaempeferol, apigenin, chrysin, luteolin (Figure 6) and their derivatives. These red wine compounds were found to be effective in the inhibition of *S. aureus* biofilm formation, where quercetin was remarkably the most active flavonoid [30]. The seeds of muscadine grape (*Vitis rotundifolia*) are rich in gallic acid, (+)-catechin and epicatechin, while the skin contains ellagic acid, myricetin, quercetin, kaempferol, and *trans*-resveratrol. [76]. Polyphenol extract from muscadine grape pomace had antibacterial activity against *S. aureus*, and at subinhibitory concentrations inhibited its biofilm formation, and at 16 × MIC it eradicated biofilms [77].

According to the results of a study by Vikram et al. flavonoids found in citrus fruit can modulate bacterial cell–cell communication, *E. coli* O157:H7 biofilm formation and *V. harveyi* virulence. Naringenin, quercetin, sinensetin and apigenin were the most active. Among the tested flavonoids, naringenin emerged as potent and possibly nonspecific inhibitor of autoinducer-mediated cell–cell signalling [29].

**Figure 7:**
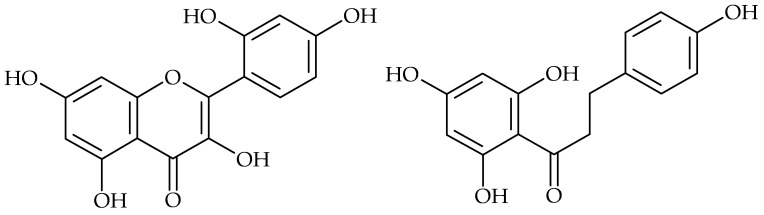
Chemical structures of morin (**left**) and phloretin (**right**).

Sivaranjani et al. explored the in vitro and in vivo antibiofilm efficacy of the flavonol morin (Figure 7) against *Listeria monocytogenes*, one of the leading foodborne pathogens. They found that morin not only inhibited biofilm production, but also reduced the virulence of *L. monocytogenes* [38]. Chalcone derivatives can also inhibit biofilm formation. This activity has been demonstrated in 2′,4′-dihydroxychalcone, 2,2′,4′-trihydroxychalcone and 2′,4′-dihydroxy-2-methoxychalcone, which inhibit *S. aureus* biofilm production [27]. Phloretin (Figure 7), an apple flavonoid, inhibited *E. coli* O157:H7 biofilm formation without inhibiting the growth of planktonic cells [31].

**Figure 8:**
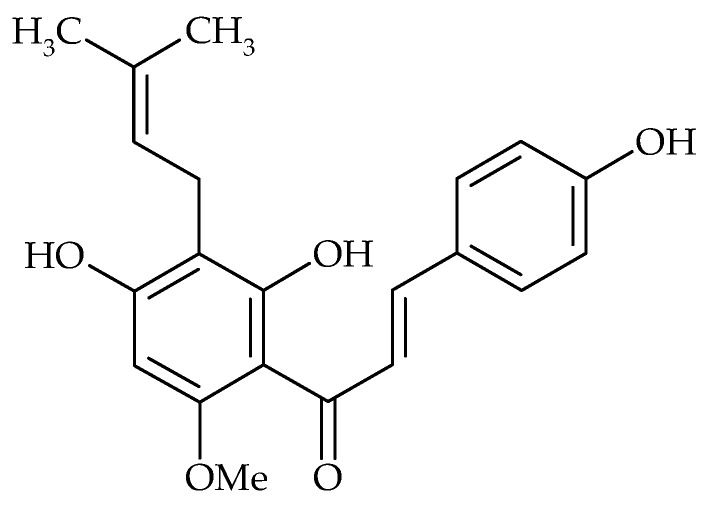
Chemical structure of xanthohumol.

Xantohumol (Figure 8), a prenylated chalconoid from *Humulus lupulus* was found to inhibit *S. aureus* adhesion and biofilm formation. It also inactivated bacteria in already formed biofilm, most likely by damaging the stability of the bacterial cytoplasmic membrane after inhibition of lipid metabolism [28].

**Figure 9:**
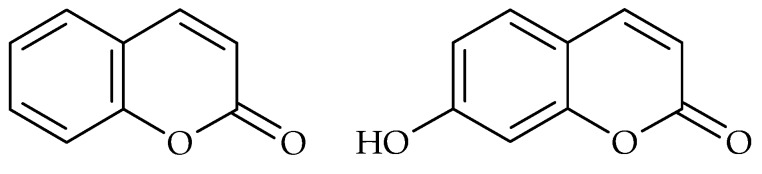
Chemical structures of coumarin (**left**) and umbelliferone (**right**).

Naturally-occurring coumarins, derivatives of 5,6-benzo-2-pyrone, display several biological activities, from photosensitizing, vasodilatating, or analgesic properties to excellent anti-inflammatory and antimicrobial activities [78]. Lee et al. examined the antibiofilm abilities of different coumarins, such as coumarin (Figure 9), coumarin-3-carboxylic acid, esculetin, 4-hydroxycoumarin, scopoletin, umbelliferone (Figure 9) and coladonin. They reported that coumarin and umbelliferone exhibited antibiofilm formation activity against enterohaemorrhagic *E. coli* O157:H7 without inhibiting planktonic cell growth. Furthermore, the biofilm of *E. coli* was inhibited by coladonin [21]. Inhibition of biofilm formation of *P. aeruginosa* was detected for esculetin, esculin, psoralen and nodakenetin [23,24,25].

Plants polyphenols could be found also in honeys of floral origin, as a result of their natural production. The well characterised Manuka honey contains mainly flavonoids and phenolic acids (for a review see [79]). Their content is closely related to the antioxidant and antimicrobial activity of honey, and they probably cooperate with the other biologically active compounds on the reported antibiofilm activity of Manuka honey [80].

## 3. Polyphenols in Periodontal Diseases and Caries

Dental biofilm ecological shift contributes to oral diseases affecting a large proportion of the human population [5]. *Streptococcus mutans* is a bacterium participating at the development of caries, thanks to its acidogenicity, aciduric properties, and an outstanding ability to produce biofilms [81,82]. Periodontal diseases, which are the major cause of tooth loss in humans, are chiefly associated with two anaerobic bacteria—*Prevotella* spp. and *Porphyromonas gingivalis* [55].

Numerous studies contain reports on polyphenols’ inhibitory effects on oral biofilm bacteria and on dental biofilm production and accumulation. Many catechin-based polyphenols, flavonoids, proanthocyanidin oligomers and some other plant-derived compounds inhibit *S. mutans* glycosyltransferase—one of the crucial virulence factors of *S. mutans* with role in synthesis of glucan polysaccharide, a major biofilm matrix component [83].

Tea polyphenols, especially EGCg, inhibited biofilm formation by *S. mutans* and reduced viability of bacteria in preformed biofilm. At subinhibitory concentrations EGCg inhibited the acidogenic and aciduric properties of this bacterium, probably by inhibition of the enzymatic activity of F1Fo-ATPase and lactate dehydrogenase, and expressed inhibition of sucrose-dependent initial attachment of *S. mutans* to surfaces [45,84]. EGCg, derived from green tea, was active also against one of the important periodontal disease agents and destroyed already established *P. gingivalis* biofilms [46] and completely inhibited the growth and adherence of *P. gingivalis* onto the buccal epithelial cells [85]. Lee and Tan observed a similar effect of EGCg also against biofilms (and other virulence factors) of *Enterococcus faecalis* [47], an agent of chronic and refractory dental canal infections [86].

Apigenin showed inhibitory activity to both glucosyltransferase and fructosyltransferase of *S. mutans* without major impact on bacterial viability and influenced the biomass and polysaccharide content of *S. mutans* biofilm [32]. Quercitrin inhibited *S. mutans* biofilm production by reducing the synthesis of both water-soluble and insoluble glucans and several virulence genes suppression [35].

*S. mutans* saccharide metabolism inhibition by several phenolic acids was detected as well. Gallic acid (and methyl gallate) had inhibitory effects on the growth of cariogenic and periodontopathic bacteria and significantly inhibited the in vitro formation of *S. mutans* biofilms [41]. Gallic acid and tannic acid at subinhibitory concentrations showed suppressive effect on *S. mutans* biofilm formation by inhibition of glucosyltransferase and fructosyltransferase [87]. However, the effect of gallic acid on biofilm formation was affected by nutrient levels, temperature, and treatment time [42].

Oligomeric proanthocyanidins, the major secondary metabolites of *Vaccinium macrocarpon* (cranberry), are further potential anticaries agents that inhibit the production of organic acids and the formation of biofilms by cariogenic bacteria [88]. Cranberry proanthocyanidins, comprised of mostly A-type oligomers of epicatechin, and flavonols (mostly quercetin glycosides) inhibited the activities of glucosyltransferases and F-ATPase, and the acid production by *S. mutans* cells. Biofilm development and acidogenicity were significantly affected by their topical application [89]. Topical applications of cranberry proanthocyanidins during biofilm formation resulted in less biomass and fewer insoluble polysaccharide formation by *S. mutans* in vitro and a significant reduction of caries incidence and less severe carious lesions in a rat dental caries model. A-type proanthocyanidin dimers and oligomers effectively diminished the synthesis of insoluble polysaccharides, and also affected bacterial glycolysis [90,91].

Several studies have evaluated the activity of grape, grape wine, grape pomace or grape seeds polyphenol extracts on biofilms produced by oral bacteria. Red wine grape (*Vitis vinifera* and *Vitis* interspecies hybrids) and its pomace phenolic extracts remarkably inhibited glucosyltransferase of *S. mutans*, as well as the glycolytic pH drop without affecting the bacterial viability, even if the anthocyanins and flavan-3-ols content were highly variable [92]. In two studies published by Furiga et al., polyphenols from red wine, grape pomace and grape seed inhibited both the formation of multi-species biofilms composed of oral bacteria (*S. mutans*, *Streptococcus sobrinus*, *Lactobacillus rhamnosus*, *Actinomyces viscosus*, *Porphyromonas gingivalis*, and *Fusobacterium nucleatum*), and the synthesis of insoluble glucan. The most effective was the grape seed extract, containing mainly catechin and epicatechin. Except to a significant antiplaque activity, the extract had synergistic effect with amine fluoride mouthwash, and showed also an important antioxidant capacity in vitro, without any bactericidal effects [93,94]. Antibacterial effect of red wine polyphenols on bacteria in the 5-species biofilm model consisting of *Actinomyces oris, F. nucleatum, Streptococcus oralis*, *S. mutans* and *Veillonella dispar* was detected by Muñoz-Gonzales et al. [95]. The powdered extract of phenolics from the pomace of Japanese wild grape (*Vitis coignetiae*) with high phenolics and flavanol content reduced adhesion of *S. mutans* to saliva-coated hydroxyapatite and biofilm formation in a dose-dependent manner, and inhibited water-soluble and water-insoluble glucans synthesis [96].

## 4. Conclusions

This review is an overview of research articles about antibiofilm activity of selected plant phenolics listed in scientific databases such as SciFinder, Science Direct, PubMed, Scopus, Web of Science, etc. Numerous plant phenolic compounds have already revealed their antimicrobial and antibiofilm activities, but the road to a clinical application form may still be long. It requires further testing—besides antimicrobial and antibiofilm effectivity—the toxicity, pharmacokinetics, pharmacodynamics, drug interactions, including classical antibiotics, and any kind of side-effects should be defined. The most feasible seems to be approval of topical application forms, which are much safer in the case of drugs with higher toxicity, and allow higher, but still safe dosages in comparison with systemic antibiotic therapy, so in the form of solutions, lotions, ointments, tinctures, gels, creams, lozenges, or suppositories, the active phenolics may come relatively soon into the practice. Inhibition of bacterial adherence to skin, mucosal and dental surfaces facilitated by topical application may also have beneficial effects in the prevention of infectious diseases, dental caries and periodontal disease. Plant phenolics-covered medical device surfaces may help in prevention of device-associated biofilm infections.

## Figures and Tables

**Table 1:** Antibiofilm activities of flavonoids, phenolic acids and tannins contained in plant extracts.

Phenolic Compound	Name of Bacteria	Antibiofilm Activity *	References
ANTHOCYANINS			
Malvidin, Petunidin, Cyanidin	*K. pneumoniae*	a,b	[20]
COUMARINS			
Coumarin	*E. coli*	a	[21,22]
	*S. aureus*	a	
	*V. anguillarum*	a	
	*E. tarda*	a	
Umbelliferone	*E. coli*	c	[21]
Esculetin	*S. aureus*	a	[23,24]
	*P. aeruginosa*	a	
Esculin	*P. aeruginosa*	a	[23]
Psoralen	*P. aeruginosa*	a	[23]
Nodakenetin	*P. aeruginosa*	a	[25]
Coladonin	*E. coli*	c	[21]
FLAVONOIDS			
Chalcone	*S. mutans*	d	[26]
2′,4′-Dihydroxychalcone	*S. aureus*	a	[27]
2,2′,4′-Trihydroxychalcone	*S. aureus*	a	[27]
2′,4′-Dihydroxy-2-methoxychalcone	*S. aureus*	a	[27]
Xanthohumol	*S. aureus*	a	[28]
Naringenin	*E. coli*	a	[29]
Hesperidin	*E. coli*	a	[29]
Neohesperidin	*E. coli*	a	[29]
	*V. harvey*	a	
Neoeriocitrin	*E. coli*	a	[29]
	*V. harvey*	a	
8-Prenylnaringenin	*S. aureus*	a	[27,28]
Apigenin	*E. coli*	a	[27,30,31,32]
	*S. aureus*	a	
	*V. harvey*	a	
	*S. mutans*	a	
Fisetin	*S. aureus*	a	[24]
Chrysin	*E. coli*	a	[30,31]
	*S. aureus*	a	
Luteolin	*E. coli* (UPEC)	a	[30,33]
	*S. aureus*	a	
Nobiletin	*E. coli*	a, e	[34]
Sinensitin	*E. coli*	a, e	[29,34]
	*V. harvey*	a, e	
Quercitrin	*S. mutans*	a	[35]
Quercetin	*E. coli*	a	[29,30,36,37]
	*S. aureus*	a	
	*V. harvey*	a	
	*S. mutans*	a	
Kaempferol	*E. coli*	a	[29,30,36]
	*S. aureus*	a	
	*V. harvey*	a	
Morin	*L. monocytogenes*	f	[38]
Phloretin	*E. coli*	g	[31]
Rutin	*E. coli, V. harvey*	a	[29]
Daidzein	*E. coli* (UPEC)	a	[31]
Genistein	*S. aureus*	a	[31,39]
	*E. coli* (UPEC)	a	
TANNINS			
Catechin	*P. aeruginosa*	a	[40]
Gallic acid	*E. coli*	a	[41,42]
	*S. mutans*	a	
Methyl gallate	*S. aureus*	h	[41,43]
	*S. mutans*	a	
(−)-Epigallocatechin gallate	*S. aureus*	a	[44,45,46,47]
	*S. epidermidis*	a	
	*S. mutans*	a	
	*P. gingivalis*	a	
	*E. faecalis*	a	
Ellagic acid	*E. coli*	a	[48]
Tannic acid	*E. coli*	a	[48]
Rosmarinic acid	*S. aureus*	a	[49,50]
1,2,3,4,6-Penta-*O-*galloyl-b-d-glucopyranose	*S. aureus*	a	[51]

* a—inhibited biofilm formation; b—inhibited EPS production; c—reductions in biofilm formation; d—sortase-specific oral biofilm inhibition; e—inhibited motility; f—in vitro and in vivo antibiofilm efficacy; g—reduced pathogenic biofilm; no harm to commensal *E. coli* K-12 biofilm formation; h—inactivated bacteria in biofilm.

## Abbreviations

MDR	Multidrug-resistant
XDR	Extremely drug-resistant
QS	Quorum sensing
EGCg	Epigallocatechin gallate
EPS	Extracellular polymeric substance
UPEC	Uropathogenic *Escherichia coli*
